# Structural damage detection using finite element model updating with evolutionary algorithms: a survey

**DOI:** 10.1007/s00521-017-3284-1

**Published:** 2017-11-22

**Authors:** Nizar Faisal Alkayem, Maosen Cao, Yufeng Zhang, Mahmoud Bayat, Zhongqing Su

**Affiliations:** 1 Department of Engineering Mechanics, Hohai University, Nanjing, 210098 Jiangsu People’s Republic of China; 2 State Key Laboratory on Safety and Health of In-Service Long-Span Bridges, Nanjing, 211112 Jiangsu People’s Republic of China; 3 Jiangsu Transportation Institute Co., Ltd., Nanjing, 211112 Jiangsu People’s Republic of China; 4 Young Researchers and Elite Club, Roudehen Branch, Islamic Azad University, Roudehen, Iran; 5 Department of Mechanical Engineering, The Hong Kong Polytechnic University, Hung Hom, Kowloon, Hong Kong, SAR

**Keywords:** Evolutionary algorithms, Finite element model updating, Structural damage detection, Dynamic characteristics, Residuals, Optimization

## Abstract

Structural damage identification based on finite element (FE) model updating has been a research direction of increasing interest over the last decade in the mechanical, civil, aerospace, etc., engineering fields. Various studies have addressed direct, sensitivity-based, probabilistic, statistical, and iterative methods for updating FE models for structural damage identification. In contrast, evolutionary algorithms (EAs) are a type of modern method for FE model updating. Structural damage identification using FE model updating by evolutionary algorithms is an active research focus in progress but lacking a comprehensive survey. In this situation, this study aims to present a review of critical aspects of structural damage identification using evolutionary algorithm-based FE model updating. First, a theoretical background including the structural damage detection problem and the various types of FE model updating approaches is illustrated. Second, the various residuals between dynamic characteristics from FE model and the corresponding physical model, used for constructing the objective function for tracking damage, are summarized. Third, concerns regarding the selection of parameters for FE model updating are investigated. Fourth, the use of evolutionary algorithms to update FE models for damage detection is examined. Fifth, a case study comparing the applications of two single-objective EAs and one multi-objective EA for FE model updating-based damage detection is presented. Finally, possible research directions for utilizing evolutionary algorithm-based FE model updating to solve damage detection problems are recommended. This study should help researchers find crucial points for further exploring theories, methods, and technologies of evolutionary algorithm-based FE model updating for structural damage detection.

## Introduction

Structural damage commonly occurs due to (a) various internal factors such as structural design faults, construction imperfections, and material shortcomings, and (b) external conditions such as earthquakes, lack of compliance with the terms of use, overloading, and environmental influences [1]. Damage can cause changes in structural dynamic properties that in turn degrade structural performance as well as safety capacity [2–4]. Hence, early damage detection to locate incipient damage provides a chance for timely structural maintenance and can guarantee structural reliability and continuing serviceability [5, 6]. Structural damage identification is usually conducted by means of non-destructive vibrational experiments that present structural dynamic characteristics such as frequency response functions (FRFs) and modal properties. These characteristics are functions of the structural physical properties and therefore can be used to portray damage based on the premise that damage alters structural physical properties, in turn causing changes in structural dynamic characteristics [7, 8].

Finite element (FE) model updating has been the subject of increasing interest in the last decade [9–12]. FE model updating can be defined as a mathematical methodology whereby a FE model is updated by gradually adjusting the model’s parameters and assumptions in such a way that the responses of the FE model progressively approach those of the counterpart real structure under investigation [13]. FE model updating provides an effective manner of structural damage detection. In an intact structure with its FE model, occurrence of damage can locally alter the structure, so that differences appear between the FE model and the structure bearing damage. Such differences can be reflected by deviations between the structural parameters of the FE model and the structure incurring damage. The deviations can be minimized by locally adjusting the FE model to bring its parameters into good agreement with the parameters of the damaged structure. Once agreement is reached, the local modification of the FE model indicates the damage. In general, FE model updating can be implemented by direct and trial-and-error methods in which the comparison is made directly between the stiffness and mass matrices of the FE model and the structure with damage [14–17]. Noticeably, these methods are somewhat inefficient in reflecting damage because the updating of the FE model might not give a reasonable physical explanation of the changes in structural characteristics [18]. Alternatively, solving FE model updating problem for structural damage identification can resort to optimization algorithms that minimize the residuals between the dynamic characteristics of the FE model and those of the damaged structure, the change in the FE model being related to structural damage.

Representative studies of damage detection based on FE model updating are summarized as follows. Sensitivity-based FE model updating for damage detection was investigated in [19–26], with the efficiency of the methods verified in various applications such as: in a simply supported beam and a concrete-filled steel tubular arch bridge [21], in four simple structures [22], in a plane frame and a 12-story shear building [23], in composite structures [24], in a reinforced concrete frame [25], and in bridge cables [26]. However, the sensitivity-based FE model updating method has some limitations: (1) it usually requires a sensitivity matrix with respect to all updating parameters, leading to expensive computation; (2) it may not be applicable to structures which contain a considerable amount of damage [27, 28]. Aside from sensitivity-based methods, statistical and probability-based FE model updating have been examined in various methods for different applications [29–40], such as Taguchi-based FE model updating for damage detection [32, 41], Bayesian framework-based FE model updating in various structures: beams [33], 2D and 3D frame structures [34], a Dowling Hall footbridge [35], aluminum hull structures [36], and IASC–ASCE benchmark building [37–39]. Despite effectiveness being addressed in these studies, statistical and probability-based FE model updating approaches have some disadvantages, such as the requirement to solve complex integrals, the need to understand the distribution of all variables, and the high computational cost [28].

Under a conventional paradigm, damage detection using FE model updating is solved as an optimization problem either directly or combined with the sensitivity-based method. This type of method depends on the strength of the optimization algorithm in handling complex and highly nonlinear FE model updating. Different optimization algorithms have been carried out to perform FE model updating for damage detection in various structures. For instance, the Nelder–Mead (NM) simplex method was employed to detect damage based on FE model updating in a simply supported beam and an asymmetrical H-shaped structure [28]; coupled local minimizers (CLM) method was utilized to detect damage based on FE model updating in a damaged reinforced concrete beam [40], a cracked beam [42], a damaged frame structure [43], and a damaged highway bridge [44]. A trust region Newton method was implemented for FE model updating of a damaged reinforced concrete frame [45], a damaged simply supported beam and a full-size precast continuous box girder bridge (Hongtang Bridge) [46], and a damaged Z24 Bridge [47]. Wang et al. [48] applied a penalty function method and a random search algorithm for damage detection based on FE model updating in a curvilinear steel box girder bridge. Other optimization methods have been used for FE model updating purposes, such as the affine scaling interior algorithm for updating a planar truss model [49], the Douglas–Reid method and Rosenbrock optimization algorithm for FE model updating of the Canonica Bridge [50], and sequential quadratic programming for the same purpose in the Bill Emerson Memorial Bridge [51].

Despite increasing uses of conventional optimization approaches in FE model updating for damage detection, they exhibit some drawbacks: (1) the gradient of the objective function is sometimes utilized to direct the optimization, entailing a process that is inefficient from the computational point of view, especially in large-scale damaged structures; (2) a solution-to-solution framework is often employed to solve the optimization problem, where a single solution is changed in each evaluation into another solution that may be better or worse. This leads to lower ability of the optimization process to detect damage, particularly when the damage patterns are distributed along the structure; (3) when solving highly nonlinear and multimodal FE model updating for damage identification problems with many local optima, there is a high probability of being stuck in local optima as well as converging to inferior solutions, that is, failure to reveal damage, especially local damage. These drawbacks easily result in low efficiency and even failure to solve optimization problems [52, 53].

Recently, EAs have been applied in various engineering disciplines such as communications engineering and informatics [54, 55], electrical engineering [56], mechanical engineering [57, 58]. Also, the use of computational intelligence for structural damage tracking [59–63] and evolutionary algorithms (EAs) as modern optimization tools to update FE models for damage detection has become a research focus [64–70]. EAs are powerful mathematical techniques that can be utilized for solving complex optimization problems of high nonlinearity, multimodal interactions, etc. Researchers have used single-objective EAs alone [71–74] or combined them with the weighted sum method to solve FE model updating problems [27, 28, 75–79]. Nevertheless, a single-objective evolutionary algorithm requires sufficient information about the problem; even so, proper distribution of solutions along the Pareto optimal front that is essential to determine the quality of the achieved solution may not be guaranteed. Moreover, selection of the combination of optimum weights is a difficult task. For this task, the commonly used trial and error is inefficient for complex FE model updating problems. For such reasons, a few researchers have applied FE model updating based on multi-objective EAs for damage detection [65–70]. Their studies have effectively demonstrated the advantage of multi-objective functions rather than conversion into single-objective functions using the weighted sum method. In contrast, the application of EAs to structural damage detection based on FE model updating is not yet well resolved.

After the introduction, the outline of this paper can be listed as: Sect. 2 defines the basic structural damage detection problem and the main FE model updating approaches. In Sect. 3, the different definitions of residuals between dynamic characteristics of FE model and the corresponding structure, used for constructing the objective function for tracking damage are presented. Section 4 details the concerns involved in selecting the FE model’s updating parameters, emphasizing the parameterization strategy for damage detection. Section 5 surveys EAs used in FE model updating for damage detection with applications. Section 6 presents a case study that evaluates the utilization of two single-objective EAs and one multi-objective EA for model updating-based structural damage identification. Section 7 summarizes future trends in the application of FE model updating based on EAs to solve damage detection problems.

## Theoretical background

### Structural damage detection problem

The simplest damage detection problem can be explained by the linear equation of motion describing the undamped free vibration paradigm as [28, 80, 81]:

1 $$\left[ M \right]\left[ {\ddot{x}} \right] + \left[ K \right]\left[ x \right] = 0,$$

where [*M*] is the mass matrix; [*K*] is the stiffness matrix; [*x*] is the displacement vector. The solution of the equation of motion can be expressed as:

2 $$\begin{aligned} x\left( t \right) & = \phi_{i} u_{i} \left( t \right), \\ u_{i} \left( t \right) & = A_{i} cos\left( {\omega_{i} t - \theta_{i} } \right), \\ \end{aligned}$$

where *ϕ*_*i*_ is the *i*th mode shape; *ω*_*i*_ is the *i*th modal frequency; *u*_*i*_ is the *i*th time variation of displacement due to harmonic excitation; *θ*_*i*_ is the *i*th phase angle; *A*_*i*_ is the *i*th constant related to the *i*th mode shape. Substituting Eq. (2) into Eq. (1) results in:

3 $$u_{i} \left( t \right)\left( { - \omega_{i}^{2} \left[ M \right]\phi_{i} + \left[ K \right]\phi_{i} } \right) = 0,$$

The non-trivial solution of Eq. (3) can be written as:

4 $$\left( {\left[ K \right] - \omega_{i}^{2} \left[ M \right]} \right)\phi_{i} = 0,$$

where Eq. (4) is called the standard eigenequation of the undamped free vibration problem.

By taking into account the continuum damage mechanics, structural damage can be defined as scalar quantities *α* ∈ [0, − 1]. The 0 value indicates the intact element, and 1 value illustrates the total failure. This can be inserted inside the FE model updating process by decreasing the global stiffness matrix to allocate damage as:

5 $$\left[ K \right]_{\text{d}} = \left[ K \right]_{\text{u}} \left( {1 - \alpha_{j} } \right),$$

where [*K*]_d_ and [*K*]_u_ are the global stiffness matrices of the damaged and intact structures, respectively; *α*_*j*_ is the damage index of the *j*th element. The problem can be transferred to an optimization problem by utilizing the following equation:

$$R_{i} \left( {\alpha_{1} ,\alpha_{2} , \ldots ,\alpha_{n} } \right) = \left\{ {\left[ K \right]_{\text{d}} - \left( {\omega_{i}^{\text{d}} } \right)^{2} \left[ M \right]} \right\}\phi_{i}^{\text{d}} , \quad r = 1, \ldots ,n,$$

6 $$F = \mathop \sum \limits_{i = 1}^{r} \left| {\left| {R_{i} } \right|} \right|^{2} ,$$

where *R*_*i*_ is the *i*th residual corresponding to *i*th vibration mode; *F* is the objective function; *r* is the number of the considered vibration modes; $\omega_{i}^{\text{d}}$ is the natural frequency of the *i*th mode.

### FE model updating methods

**Fig. 1:**
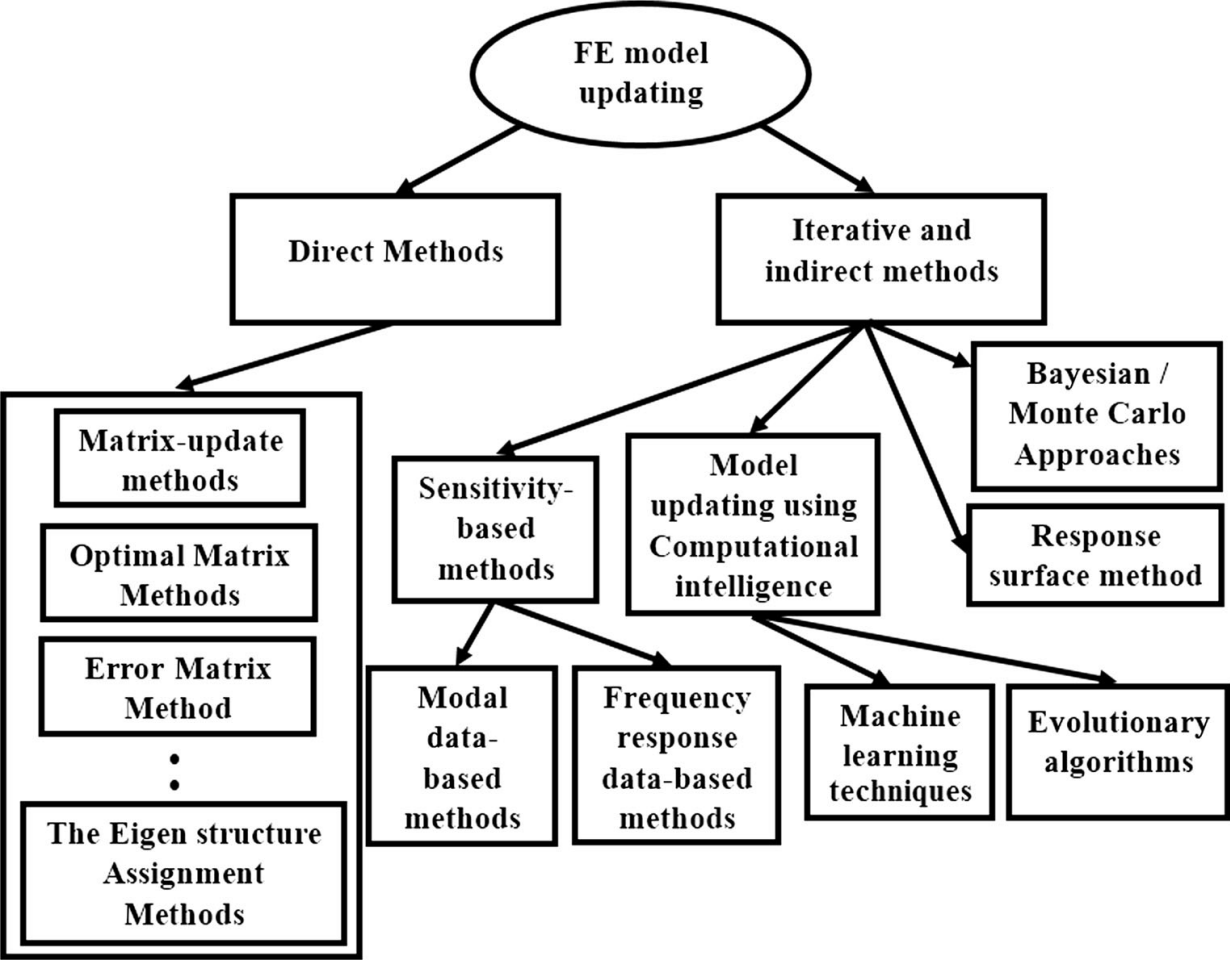
FE model updating approaches

As it has been explained, the FE model updating process is a mathematical procedure by which an initial FE model of an intact structure is amended to achieve a good agreement between the damaged structure and its FE model [28]. Various techniques have been developed for FE model updating purpose. Those methods were surveyed by Mottershead and Friswell [18, 81] and Marwala [28] and can be categorized into two main classes as: (1) direct methods; (2) iterative and indirect methods as it is shown in Fig. 1.

Direct methods use modal characteristics to update the FE model. They are considered as accurate methods and efficient from computational point of view. Moreover, they do not require updating parameters to be handled. Several direct model updating techniques were developed by various researchers such as the matrix-update [82], optimal matrix, error matrix [81], eigenstructure assignment [83] methods. The advantages of model updating using direct methods are:

- Direct methods do not apply iterative paradigms, so they insure the accurate and convergence to the exact solution with computational efficiency.
- They do not consider updating physical parameters of structures.
- The updated FE model can reflect the exact measured quantities.

Although direct methods are efficient, they have many drawbacks making them non-reliable as [28]:

- They require accurate measurements, and they are highly sensitive for noise.
- Measured and calculated responses need to be equal in size.
- Direct method may produce unrealistic representation of elements along the FE mesh. In other words, loss of symmetry may appear in model’s matrices.
- Possibility of losing the connectivity of the structure and the updated model’s matrices are fully populated.

Because of the above-mentioned difficulties, direct methods are not applicable for damage detection purposes. Hence, iterative and indirect approaches come into picture. Those methods can be summarized as:

- *Sensitivity*-*based methods* They consider the measured responses as alterations of some design data derived from the initial FE model of the intact structure and the optimization problem is formulated using a penalty function approach [28, 81]. Using this concept, the measured responses must be near the calculated data deduced from the initial FE model making the sensitivity methods applicable only when changes in the real structure are within a small scale. Hence, they can be implemented just in the case of structures with minor damage. The main philosophy of sensitivity-based methods is to calculate derivatives of modal characteristics or frequency response data that makes the overall procedure computationally expensive [81, 84]. Representative researches for using sensitivity method for structural damage detection using FE model updating can be seen in the work of Sarvi et al. [85] and Yu et al. [86].
- *The response*-*surface method* (*RSM*) The RSM is a statistical approach that develops a correlation between a set of predetermined design variables and their respective responses commonly as polynomial functions. In FE model updating, RSM can deduce the best response that matches the least variation between the initial FE model and the measured responses [28, 46, 87]. This makes the RSM easy to implement with good computational efficiency. Moreover, RSM can provide effective solution for complex model updating problems. Examples of application of RSM in FE model updating can be observed in [46, 87]. The disadvantage of RSM for structural damage detection using model updating is that it applies statistical approximations with unknown parameters that may not reflect the real damaged locations along the structure [28]. Moreover, in large-scale structures, the FE model updating using RSM still needs more research [87].
- *The Bayesian*–*Monte Carlo method* The Bayesian method is a modern FE model updating technique influenced by the Bayes’ theorem in which by considering a set of data with a probability distribution it can reflect the probability distribution of a model [28]. Bayesian methods are usually solved by Monte Carlo approaches. They outcome accurate deductions without over fitting. Moreover, the parameter estimation process is easy to implement with sufficient physical explanations of the results [33]. A detailed explanation of Bayesian–Monte Carlo model updating methodology can be referenced by many research papers such as [33, 34, 37]. The Bayesian–Monte Carlo model updating was used for damage detection purposes. They were implemented successfully by Kurata et al. [88] for damage identification in plate-type structures as well as Lam and Yang [90] for damage tracking in steel towers. Sohn and Law [89] developed a Bayesian approach for damage inference in a reinforced concrete bridge structure with superior results. Nevertheless, the Bayesian–Monte Carlo model updating methods have tangible difficulties such as the requirements of solving complex integrals which lead to high computational cost. Moreover, the initial knowledge of intervals and distributions of updating parameters must be known in advance [28, 33].
- *Computational intelligence model updating techniques* Computational intelligence techniques are utilized for model updating due to the fact that model updating is ultimately an optimization problem in which the structural physical parameters are updated to achieve relative matching between the FE model of the healthy structure and the structure bearing damage [28]. The uncertainty in updating parameters influenced the use of computational intelligence for model updating purposes. Main computational intelligence techniques include machine learning and evolutionary algorithms. Representative researches of machine learning-based model updating can be observed in the work of Fei et al. [91] in which they developed ANN models for model updating of nonlinear beam elements using frequency response data with efficient outcomes. Zapico et al. [92] implemented ANNs for the FE model updating of a small-scale frame using the natural frequencies as dynamic responses. Results demonstrated accurate updating when clarified with experiments. Zhu and Zhang [93] utilized support vector machines for FE model updating of 164 FE model of an aircraft, and results showed precise matching between natural frequencies of the updated model and the real structure. Other interesting implementation of machine learning techniques can be seen in [28].
- *EAs* Other computational intelligence techniques are EAs which are efficient mathematical approaches able to solve complex optimization problems of high nonlinearity, multimodal interactions, etc. As it has been shown in the introduction, EAs have been effectively used in cases of complex FE model updating for damage detection. Besides the conventional optimization methods, EAs do not require the calculation of the objective function gradients, a process that is not suitable for large-scale structure with major damage. Moreover, EAs are population-based optimization approaches that improve a set of possible solutions rather than a solution-to-solution framework [52, 53]. These features enhance the ability of detecting damage, especially when damage locations are distributed along the structure. Furthermore, EAs have stronger ability to overcome optimization problems with many local optima which is a great issue that appears when solving highly nonlinear and multimodal FE model updating for damage identification problems. Such feature gives reliability to EAs, especially when damage is located along a structure under consideration [28]. The above-mentioned advantages influenced researchers worldwide to apply EAs for FE model updating alone or for damage identification purpose.

**Fig. 2:**
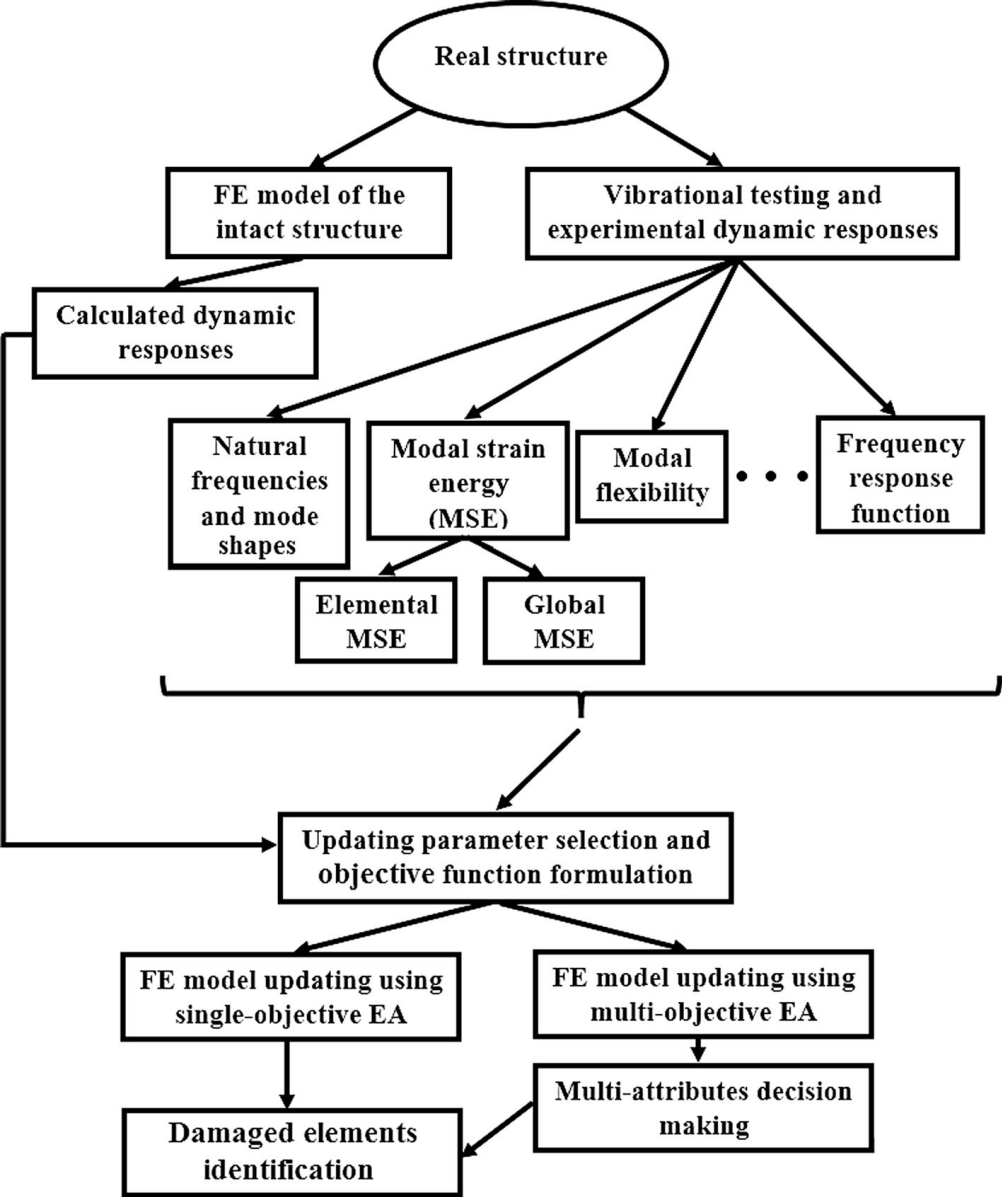
The organization of structural damage detection using FE model updating with EAs

To summarize, it is observed that the modern FE model updating approaches for structural damage assessment purpose are the Bayesian–Monte Carlo-based, machine learning-based and EAs-based methods. In this paper, our aim is to give a complete overview about critical aspect and main methodology of damage detection in structures using FE model updating with EAs as it is shown in Fig. 2.

## Residuals of dynamic characteristics: damage portrayal

In structural damage detection based on FE model updating, the theoretical core is to formulate residuals between dynamic characteristics of the initial FE model that reflect the undamaged structure and the structure with damage. The residuals reflect the deviation of the initial FE model from the structure with damage, suitable for formulating the objective function for damage characterization. A valid residual is highly dependent on the proper selection of dynamic characteristics. In what follows, we review the representative residuals used in FE model updating for structural damage detection. Those residuals can be classified in terms of dynamic characteristics: natural frequencies, mode shapes, FRFs, modal flexibility, and modal strain energy.

### Natural frequencies and mode shapes

Natural frequencies and modal vectors are fundamental dynamic characteristics of structures. These characteristics can provide general information about damage-caused changes in structural dynamic properties. Hence, natural frequencies and mode shapes can be utilized to formulate residuals for use in FE model updating. The residual of natural frequency [94, 95], Ω*R*, can be written as

7 $$\Omega R = \sum\limits_{{i = 1}}^{N} {\gamma _{i} } \times \left( {\frac{{\omega _{i}^{{\text{I}}} - \omega _{i}^{{\text{D}}} }}{{\omega _{i}^{{\text{D}}} }}} \right)^{2} ,$$

where $\omega_{i}^{\text{I}}$ and $\omega_{i}^{\text{D}}$ are the *i*th natural frequencies generated from the initial FE model of the undamaged structure and from the damaged structure, respectively; *γ*_*i*_ is a weighting factor that indicates the relative contribution of *i*th natural frequency to Ω*R*. *γ*_*i*_ is usually set to 1 for the first natural frequencies, implying that the first natural frequencies have greater contribution or importance than the latter ones in reflecting changes of structural dynamic properties between the FE model and the structure.

The residual of mode shape is formulated based on the correlation between the modal vectors of the initial FE model of the intact structure and the structure involving damage. The most typical correlation is the modal assurance criterion (MAC) [94]. The MAC is a scalar quantity that measures the consistency between a reference modal vector and another measured modal vector. The MAC can be defined as

8 $${\text{MAC}}\left( {\left\{ {\varphi_{\text{I}} } \right\},\left\{ {\varphi_{\text{D}} } \right\}} \right) = \frac{{\left| {\left\{ {\varphi_{\text{I}}^{*} } \right\}^{\text{T}} \left\{ {\varphi_{\text{D}} } \right\}} \right|^{2} }}{{\left\{ {\varphi_{\text{I}}^{*} } \right\}^{\text{T}} \left\{ {\varphi_{\text{I}} } \right\}\left\{ {\varphi_{\text{D}}^{*} } \right\}^{\text{T}} \left\{ {\varphi_{\text{D}} } \right\}}} ,$$

where {*φ*} is the modal vector; I labels the initial FE model of the undamaged structure; D denotes the damaged structure; T denotes the manipulation of transpose; and * represents the complex conjugate. If the MAC value is equal to 1, there is complete consistency between the modal vectors of the FE model and the structure, and a 0 value addresses the entire inconsistency.

Several mode shape residuals have been developed using the MAC by researchers [28, 65, 95]:

9 $$\varphi R_{1} = \mathop \sum \limits_{i = 1}^{N} \beta_{i} \times \left( {1 - {\text{MAC}}_{i} \left( {\left\{ {\varphi_{i}^{\text{I}} } \right\},\left\{ {\varphi_{i}^{\text{D}} } \right\}} \right)} \right),$$

10 $$\varphi R_{2} = \mathop \sum \limits_{i = 1}^{N} \beta_{i} \times \left( {1 - {\text{diag}}\left( {{\text{MAC}}_{i} \left( {\left\{ {\varphi_{i}^{\text{I}} } \right\},\left\{ {\varphi_{i}^{\text{D}} } \right\}} \right)} \right)} \right),$$

11 $$\varphi R_{3} = \mathop \sum \limits_{i = 1}^{N} \beta_{i} \times \frac{{\left( {1 - \sqrt {{\text{MAC}}_{i} } } \right)^{2} }}{{{\text{MAC}}_{i} }},$$

where *φR* is the mode shape residual; MAC_*i*_ is the MAC value corresponding to the *i*th mode shape; *φ* ^I^ and *φ* ^D^ denote the modal vectors obtained from the initial FE model of the undamaged structure and those from the damaged structure; diag(MAC) represents the *i*th diagonal element of the MAC matrix; *β*_*i*_ is a weighting factor giving the relative importance of MAC_*i*_.

Perera and Ruiz [95] developed a modified total modal assurance criterion (MTMAC) by fusing natural frequencies and the modal vectors into one expression. The MTMAC can be expressed by

12 $${\text{MTMAC}}_{i} = \frac{{{\text{MAC}}\left( {\left\{ {\varphi_{i}^{\text{I}} } \right\},\left\{ {\varphi_{i}^{\text{D}} } \right\}} \right)}}{{1 + \left| { \omega_{i}^{{{\text{D}}2}} - \omega_{i}^{{{\text{I}}2}} /\omega_{i}^{{{\text{D}}2}} + \omega_{i}^{{{\text{I}}2}} } \right| }}$$

The residual depending on the MTMAC can be given by

13 $${\text{MTR}} = 1 - {\text{MTMAC}} = 1 - \mathop \prod \limits_{i = 1}^{N} {\text{MTMAC}}_{i}$$

where MTR is the modified total modal assurance criterion residual; and *N* is the number of mode shapes.

### FRFs

Model updating using FRF data has been reported by various researchers [96–102]. The FRF residual is established on the frequency domain assurance criterion (FDAC) [103]

14 $${\text{FDAC}}\left( {\omega_{i}^{\text{I}} ,\omega_{j}^{\text{D}} } \right) = \frac{{H_{i}^{{{\text{I}}^{\text{T}} }} .H_{j}^{\text{D}} }}{{H_{i}^{\text{I}} H_{j}^{\text{D}} }} ,$$

where $H_{i}^{\text{I}}$ is the *i*th FRF generated from the initial FE model and $H_{j}^{\text{D}}$ is the *j*th FRF from the damaged structure.

The FDAC may vary within the interval [− 1, 1]: the value 1 indicates complete consistency between the FRF from the initial FE model and that from the damage structure, and a value greater than 0 implies that the two FRFs are in the same phase.

A modified FDAC with a similar form to the MAC was proposed by Yan and Golinval [102]

15 $${\text{FDAC}}\left( {\omega_{i}^{\text{I}} ,\omega_{j}^{\text{D}} } \right) = \frac{{\left( {H_{i}^{{{\text{I}}^{\text{T}} }} .H_{j}^{\text{D}} } \right)\left| {H_{i}^{{{\text{I}}^{\text{T}} }} .H_{j}^{\text{D}} } \right|}}{{\left( {H_{i}^{{{\text{I}}^{\text{T}} }} H_{i}^{\text{I}} } \right)\left( {H_{j}^{{{\text{D}}^{\text{T}} }} H_{j}^{\text{D}} } \right)}}$$

The FDAC was further modified [102, 103] to be suitable for incomplete measured FRFs, leading to the simplified frequency domain assurance criterion (SFDAC). This criterion can only be used on the set of natural frequencies obtained from damaged structure as expressed in the following

16 $${\text{SFDAC}}_{i} = \frac{{\left( {H_{i}^{{{\text{I}}^{\text{T}} }} .H_{i}^{\text{D}} } \right)\left| {H_{i}^{{{\text{I}}^{\text{T}} }} .H_{i}^{\text{D}} } \right|}}{{\left( {H_{i}^{{{\text{I}}^{\text{T}} }} H_{i}^{\text{I}} } \right)\left( {H_{i}^{{{\text{D}}^{\text{T}} }} H_{i}^{\text{D}} } \right)}},$$

where *i* = 1, …, *N*_D_; *N*_D_ is the number of frequencies related to the structure with damage; SFDAC is a vector containing scalars ∈ [− 1, 1].

Finally, the mean value of the SFDAC (Eq. 16) can be used to develop the FRF residual as

17 $$\overline{\text{SFDAC}} = \frac{1}{{N_{\text{D}} }}\mathop \sum \limits_{i = 1}^{{N_{\text{D}} }} {\text{SFDAC}}_{i} ,$$

18 $${\text{FRFR}} = 1 - \overline{\text{SFDAC}} = 1 - \frac{1}{{N_{\text{D}} }}\mathop \sum \limits_{i = 1}^{{N_{\text{D}} }} {\text{SFDAC}}_{i} ,$$

where FRFR is the FRF residual; *N*_D_ is the number of frequencies considered.

From Eq. (17), the value of 1 of $\overline{\text{SFDAC}}$ means that there is complete agreement between the initial model predicted and the FRF obtained from damaged structure, leading to the minimum value of FRFR equal to 0, which can be more convenient for structural damage detection based on the FE model updating procedure.

### Modal flexibility

The modal flexibility parameter has been reported as a sensitive parameter for identification of local damage in structures [104–107]. Modal flexibility can be defined by employing natural frequencies and modal vectors. To derive the modal flexibility residual, we begin with the non-damped free vibration equation [11].

19 $$\left[ M \right]\ddot{x} + \left[ K \right]x = 0,$$

where [*M*] and [*K*] are the mass matrix and stiffness matrix, respectively; *x* is the displacement. The solution of the eigenvalue problem can be written as

20 $$\left[ \varphi \right]^{\text{T}} \left[ K \right]\left[ \varphi \right] = \left[ \lambda \right],\quad \left[ \varphi \right]^{\text{T}} \left[ M \right]\left[ \varphi \right] = \left[ I \right],$$

where [*φ*] denotes the eigenvector matrix; [*λ*] refers to the diagonal matrix containing the squares of the natural frequencies; [*I*] is the unity matrix. From Eq. (20), we can write

21 $$\left[ K \right] = \left[ \varphi \right]^{{ - {\text{T}}}} \left[ \lambda \right]\left[ \varphi \right]^{ - 1} = \left( {\left[ \varphi \right]\left[ \lambda \right]^{ - 1} \left[ \varphi \right]^{\text{T}} } \right)^{ - 1}$$

The modal flexibility matrix [*F*] can be derived from the inverse of the stiffness matrix [*K*] as

22 $$\left[ F \right] = \left[ K \right]^{ - 1} = \left[ \varphi \right]\left[ \lambda \right]^{ - 1} \left[ \varphi \right]^{\text{T}} = \mathop \sum \limits_{i = 1}^{N} \frac{1}{{\omega_{i}^{2} }}\left[ {\varphi_{i} } \right]\left[ {\varphi_{i} } \right]^{\text{T}} ,$$

where *ω*_*i*_ is the natural frequency corresponding to the *i*th mode shape number.

Usually, modal vectors and natural frequencies are not obtainable for all degrees of freedom. For that reason, it is essential to divide the modal flexibility matrix into two sub-matrices. The first sub-matrix is related to measured mode shapes and the second is related to the remaining unmeasured mode shapes, as follows:

23 $$\left[ F \right] = \left[ {F_{\text{ms}} } \right] + \left[ {F_{\text{um}} } \right] = \left[ {\varphi_{\text{ms}} } \right]\left[ {\lambda_{\text{ms}} } \right]^{ - 1} \left[ {\varphi_{\text{ms}} } \right]^{\text{T}} + \left[ {\varphi_{\text{um}} } \right]\left[ {\lambda_{\text{um}} } \right]^{ - 1} \left[ {\varphi_{\text{um}} } \right]^{\text{T}} ,$$

where *F*_ms_ and *F*_um_ are the modal flexibility matrices for measured and unmeasured mode shapes; *φ*_ms_ and *φ*_us_ are the measured and unmeasured modal vectors; *λ*_ms_ and *λ*_um_ are the squares of the measured and unmeasured natural frequencies, respectively.

The next procedure is to normalize the modal vectors using the mass matrix, in order to overcome the difficulty of incomplete mode shapes due to ambient vibration experiments. Jaishi et al. [21] used the Guyan mass matrix reduction method [108] that ignores the inertial forces at unmeasured degrees of freedom. This presumption helps to utilize only the first set of natural frequencies. Normalization of the modal vectors using the Guyan method can be expressed as

24 $$\phi_{ij} = \frac{{\varphi_{ij} }}{{\sqrt {\left\{ {\varphi_{j} } \right\}^{\text{T}} \left[ M \right]\left\{ {\varphi_{j} } \right\}} }},$$

where *ϕ*_*ij*_ is the value of the normalized modal vector corresponding to the *i*th mode shape and the *j*th degree of freedom, respectively. Normalization of the modal vectors in cases of a diagonal mass matrix can be written as

25 $$\phi_{ij} = \frac{{\varphi_{ij} }}{{\sqrt {\mathop \sum \nolimits_{k = 1}^{N} m_{k} \varphi_{kj}^{2} } }},$$

Finally, the deflection vector *v*_*i*_ under a uniformly distributed unit load is defined in Eq. (20) to form the modal flexibility residual as in Eq. (27).

26 $$v_{i} = \mathop \sum \limits_{k = 1}^{{N_{\text{mv}} }} \frac{{\left( {\phi_{ik} } \right)\mathop \sum \nolimits_{j = 1}^{{N_{\text{d}} }} \left( {\phi_{kj} } \right)}}{{\omega_{k}^{2} }} ,$$

27 $${\text{RF}}^{2} = \mathop \sum \limits_{i = 1}^{{N_{\text{d}} }} \left( {\frac{{v_{i}^{\text{I}} - v_{i}^{\text{D}} }}{{v_{i}^{\text{E}} }}} \right)^{2} ,$$

where RF is the modal flexibility residual; *N*_mv_ and *N*_d_ are the numbers of measured degrees of freedom and the mode shapes, respectively; $v_{i}^{\text{I}}$ and $v_{i}^{\text{D}}$ are the deflection vectors of the initial FE model and the damaged structure under a uniformly distributed unit load, respectively.

To develop the modal flexibility residual without using reduction methods, a modified modal assurance criterion called the ‘modal assurance criterion for modal flexibility’ (MACF) was introduced by Perera and Ruiz [95] as

28 $${\text{MACF}}_{i} = \frac{{\left| {\left\{ {F_{i}^{\text{I}} } \right\}^{\text{T}} \left\{ {F_{i}^{\text{D}} } \right\}} \right|^{2} }}{{\left( {\left\{ {F_{i}^{\text{I}} } \right\}^{\text{T}} \left\{ {F_{i}^{\text{I}} } \right\}\left\{ {F_{i}^{\text{D}} } \right\}^{\text{T}} \left\{ {F_{i}^{\text{D}} } \right\}} \right)}},$$

where $\left\{ {F_{i}^{\text{I}} } \right\}$ and $\left\{ {F_{i}^{\text{D}} } \right\}$ are the initial FE model predicted and the damaged structure’s modal flexibility vectors corresponding to the *i*th mode shape number.

The modal flexibility residual RF can be written utilizing the MACF as

29 $${\text{RF}} = 1 - {\text{MACF}} = 1 - \mathop \prod \limits_{i = 1}^{N} {\text{MACF}}_{i}$$

The main benefit of using the modified MACF when developing an objective function is that the objective function values are bounded by an interval with limits of 0 and 1. Furthermore, there is no need to use a mass reduction normalization method, an advantage that can lead to easier application of the FE model updating procedure.

### Modal strain energy

Various studies have shown the efficiency of using modal strain energy (MSE) as a sensitive indicator for damage [65, 109–111]. The ability of MSE to detect minor damage in complex structures is superior to that of other modal analyses such as modal vectors and natural frequencies [112–115]. Moreover, MSE-based damage indicators can be successfully implemented for FE model updating of 3D structures such as buildings [65, 98, 116–118]. Structural damage can be explained by means of the reduction in stiffness [119]. Although reduction of stiffness may not explain all damage cases, it can represent cases in which the damage varies linearly. For this purpose, two methodologies for developing MSE residuals can be described as the elemental MSE residual and the global strain energy residual.

#### Global MSE

Jaishi and Ren [70] described a MSE damage indicator in which the MSE residual is represented by employing the global stiffness matrix of the structure. Hence, the FE model predicted MSE for the undamaged structure and the MSE of the structure bearing damage corresponding to the *i*th mode shape are defined as

30 $${\text{MSE}}_{i}^{\text{I}} = \frac{1}{2}\left( {\varphi_{i}^{\text{I}} } \right)^{\text{T}} K\left( {\varphi_{i}^{\text{I}} } \right),$$

31 $${\text{MSE}}_{i}^{\text{D}} = \frac{1}{2}\left( {\varphi_{i}^{\text{D}} } \right)^{\text{T}} K\left( {\varphi_{i}^{\text{D}} } \right),$$

where ${\text{MSE}}_{i}^{\text{I}}$ and ${\text{MSE}}_{i}^{\text{D}}$ imply the initial FE model MSE and the damaged structural MSE corresponding to the *i*th mode shape, respectively; $\varphi_{i}^{\text{I}}$ and $\varphi_{i}^{\text{D}}$ denote the modal vectors of the initial FE model and the modal vectors of the structure bearing brooking damage corresponding to *i*th mode shape, respectively; and *K* is the global stiffness matrix of the structure.

The global MSE residual for FE model updating can be defined as the summation of the square errors between MSE^I^ and MSE^D^ as

32 $${\text{MSEGR}} = \mathop \sum \limits_{i = 1}^{V} \left( {\frac{{\left( {\varphi_{i}^{\text{I}} } \right)^{\text{T}} K\left( {\varphi_{i}^{\text{I}} } \right) - \left( {\varphi_{i}^{\text{D}} } \right)^{\text{T}} K\left( {\varphi_{i}^{\text{D}} } \right)}}{{\left( {\varphi_{i}^{\text{D}} } \right)^{\text{T}} K\left( {\varphi_{i}^{\text{D}} } \right)}}} \right)^{2} ,$$

where MSEGR is the global MSE residual and *V* is the total number of modal vectors. The normalization between the initial FE model’s predicted modal vectors and the experimentally obtained modal vectors for the damaged structure should be consistent. Also, the experimental modal vectors must be consistent with the degrees of freedom of the FE model. To overcome such difficulties, Jaishi and Ren [70] utilized the modal scale factor (MSF) method originally proposed by Allemang and Brown [120, 121], by multiplying the incomplete modal vectors by the MSF, where the MSF can be stated as

33 $${\text{MSF}}_{i} = \frac{{\left( {\varphi_{i}^{\text{I}} } \right)^{\text{T}} \left( {\varphi_{i}^{\text{D}} } \right)}}{{\left( {\varphi_{i}^{\text{D}} } \right)^{\text{T}} \left( {\varphi_{i}^{\text{D}} } \right)}}$$

The subsequent task is to expand the experimental modal vectors by using one of several modal expansion techniques. Jaishi and Ren [70] used the expansion method of Lipkins and Vandeurzen [122], in which the experimental modal vectors are considered as a linear combination of the modal vectors predicted by the initial FE model and a transformation matrix T, as shown in Eq. (34).

34 $$\left[ {\varphi^{\text{E}} } \right] = \left[ {\begin{array}{*{20}l} {\left[ {\varphi_{1}^{\text{D}} } \right]_{n \times v } } \hfill \\ {\left[ {\varphi_{2}^{\text{D}} } \right]_{{\left( {N - n} \right) \times v}} } \hfill \\ \end{array} } \right] = \left[ {\begin{array}{*{20}l} {\left[ {\varphi_{1}^{\text{I}} } \right]_{n \times u } } \hfill \\ {\left[ {\varphi_{2}^{\text{I}} } \right]_{{\left( {N - n} \right) \times u}} } \hfill \\ \end{array} } \right] \times \left[ T \right]_{v \times u} ,$$

35 $$\left[ T \right] = \left( {\left[ {\varphi_{1}^{\text{I}} } \right]^{\text{T}} \left[ {\varphi_{1}^{\text{I}} } \right]} \right)^{ - 1} \left( {\left[ {\varphi_{1}^{\text{I}} } \right]^{\text{T}} \left[ {\varphi_{1}^{\text{D}} } \right]} \right)$$

where *φ* ^D^ is the expanded experimental modal vectors obtained from the structure suffering damage; *N* is the number of degrees of freedom; *n* is the number of experimentally obtained degrees of freedom; *v* and *u* are the number of the obtained modal vectors and the added modal vectors, respectively; T is the transformation matrix.

#### Elemental MSE

The elemental MSE damage indicator was implemented by Cha and Buyukozturk [65] and can be stated as the summation of inner products between the square of the *i*th modal vector *φ*_*i*_ and the element stiffness matrix *K*_*j*_ corresponding to the *j*th element. The MSE predicted by the initial FE model and the experimentally calculated MSE of the damaged structure can be shown as

36 $${\text{MSE}}_{ij}^{\text{I}} = \left( {\varphi_{i}^{\text{I}} } \right)^{\text{T}} K_{j} \left( {\varphi_{i}^{\text{I}} } \right),$$

37 $${\text{MSE}}_{ij}^{\text{D}} = \left( {\varphi_{i}^{\text{D}} } \right)^{\text{T}} K_{j} \left( {\varphi_{i}^{\text{D}} } \right),$$

where ${\text{MSE}}_{ij}^{\text{I}}$ and ${\text{MSE}}_{ij}^{\text{D}}$ are the estimated MSE of the undamaged structure FE model and the structural MSE in the case of damage corresponding to the *i*th modal vector and the *j*th elemental stiffness matrix.

The elemental MSE residual for damage detection via FE model updating can be expressed as the summation of the absolute errors between MSE^I^ and MSE^D^ as

38 $${\text{MSEER}} = \mathop \sum \limits_{i = 1}^{V} \mathop \sum \limits_{j = 1}^{L} \left| {\left( {\varphi_{i}^{\text{I}} } \right)^{\text{T}} K_{j} \left( {\varphi_{i}^{\text{I}} } \right) - \left( {\varphi_{i}^{\text{D}} } \right)^{\text{T}} K_{j} \left( {\varphi_{i}^{\text{D}} } \right)} \right|,$$

where MSEER is the elemental MSE residual; *V* and *L* are the total number of modal vectors and the total number of model elements, respectively.

## Selection of updating parameters: damage parameterization

Structural damage is usually defined as the change in various mechanical parameters of a structure [1]. This change can be directly employed to detect damage locations by connecting the FE model elements and the structural parameters within a FE model updating procedure. The updating parameters are the set of underlying parameters in the FE model that can be varied to update the initial model [70, 94]. Selection of the updating parameters associated with the FE model updating procedure is a crucial issue that can determine the quality of the updated model, especially when it is used for detection of structural damage. Usually, the updated parameters are selected depending on the type of structure being considered and with understanding of the overall parameters used to model it. Several key points must be considered before selection of the updating parameters. First, there should be a focus on the locations where damage is likely to occur. Next, formulation of the objective function for the FE model updating problem must take into account the residuals that are sensitive to the selected updating parameters. Finally, the set of updating parameters should be as small as possible, to eliminate unnecessary parameters and reduce computation cost [123, 124].

Various studies have been concerned with parameterization methods. The basic and simplest strategy is to define scalar multipliers associated with the mass, stiffness, and damping matrices [118], as is shown in Eqs. [39–41].

39 $$M = M^{\text{I}} + \alpha_{1} M_{1} + \alpha_{2} M_{2} + \cdots + \alpha_{m} M_{m} ,$$

40 $$K = K^{\text{I}} + \beta_{1} K_{1} + \beta_{2} K_{2} + \cdots + \beta_{k} K_{k} ,$$

41 $$C = C^{\text{I}} + \gamma_{1} C_{1} + \gamma_{2} C_{2} + \cdots + \gamma_{c} C_{c} ,$$

where *M*, *K*, and *C* are the mass, stiffness, and damping matrices, respectively; I denotes the initial FE model’s matrices; *m*, *k*, and *c* are the chosen mass, stiffness, and damping updating parameter numbers; *α*, *β*, and *γ* are the non-dimensional multipliers. By using this strategy, the updating parameters can be applied to sub-structures containing elements that share common features or to individual elements that are scattered along the FE mesh and must be updated for special reasons strongly related to the structure under consideration.

Another parameterization strategy employs the direct material and geometrical properties of the structure [125]. Material properties such as Young’s modulus of elasticity *E* and mass density *ρ* are usually chosen as updating parameters to indicate the damage along the model’s elements. Moreover, the stiffness and mass matrices are proportional to Young’s modulus and mass density, respectively, and that makes the updating procedure easier to implement. Other parameters, geometrical parameters that are highly linked to structural damage, can be chosen, such as the element cross-sectional area *A* and the thickness of the element *t*. Mottershead et al. [81] recommended that *E* and *ρ* should not be chosen independently because that can lead to identical eigenvalue sensitivities as well as *A* and *t* because of the difficulty in physical interpretation. The above-mentioned parameters can be useful updating parameters because they are strongly linked to the overall elements along the FE mesh and the perturbation of those parameters can effectively reflect damage cases.

If we choose an updating parameter as *x*, a normalized factor *α* ∈ [0, 1] is commonly used to measure the relative change between the initial updating parameter *x* ^0^ and the updated parameter *x*. The change in this factor *α* can reflect existing damage that has already occurred in different locations (elements) in the structure. The relative change of the selected updating parameter can be expressed as

42 $$x_{i} = x^{0} \left( {1 - \alpha_{i} } \right),$$

where *α*_*i*_ is the normalization factor corresponding to the *x*_*i*_ updating parameter related to the *i*th FE model element. Hence, the updated stiffness and mass matrices can be written as

43 $$M_{i}^{\text{U}} = M_{i}^{\text{I}} + \Delta M_{i} ,$$

44 $$K_{i}^{\text{U}} = K_{i}^{\text{I}} + \Delta K_{i} ,$$

where $M_{i}^{\text{U}}$ and $K_{i}^{\text{U}}$ are the updated elemental mass and stiffness matrices, respectively; $M_{i}^{\text{I}}$ and $K_{i}^{\text{I}}$ are the initial elemental mass and stiffness matrices, respectively; ∆*M*_*i*_ and ∆*K*_*i*_ are the changes in mass and stiffness matrices, respectively, that can be calculated as

45 $$\Delta M_{i} = x_{i} .M_{i}^{\text{I}} ,\quad \Delta K_{i} = x_{i} .K_{i}^{\text{I}}$$

The FE modeling assumptions can notably affect the accuracy of the model. Several assumptions are usually made to facilitate the modeling process. One of the most general assumptions is to consider links and boundaries between elements to be rigid, although that is not true in practice, especially when the damage occurs. Flexible joints strongly reflect structural damage along the FE model’s mesh and are less likely to be rigid. One effective strategy is the method of offset nodes in which the FE nodes’ dimensions are to be varied to simulate the flexibility of the joints, a strategy that can improve the accuracy of the model [126].

Another effective parameterization strategy introduced by Ahmadian et al. [127, 128] is the generic elements method. This method implements the procedure of updating the stiffness and mass matrices by adjusting the eigenvectors and eigenvalues of the individual elements or sub-structures. In this method, the eigenvectors of an element having $\left( {K_{0}^{e} , M_{0}^{e} } \right)$ stiffness and mass matrices with the number of degrees of freedom *d* is less than or equal to six can be written as

46 $$\Phi _{0}^{e} = \left[ {\phi_{1} ,\phi_{2} , \ldots ,\phi_{d} |\phi_{d + 1} , \ldots ,\phi_{r} } \right] = \left[ {\Phi _{\text{R}} ,\Phi _{\text{S}} } \right],$$

and

47 $$\left( {\Phi _{0}^{e} } \right)^{\text{T}} M_{0}^{e}\Phi _{0}^{e} = I,\quad \left( {\Phi _{0}^{e} } \right)^{\text{T}} K_{0}^{e}\Phi _{0}^{e} =\Lambda ,$$

where *R* and *S* are the rigid-body and strain, respectively; $\Phi _{0}^{e}$ is the element eigenvector; Λ is the element eigenvalue matrix.

For updating the FE model, Ahmadian et al. [127, 128] assumed that an initial model to be updated had the elemental mass and stiffness matrices $\left( {K_{0}^{e} , M_{0}^{e} } \right)$ and corresponding eigenvectors and eigenvalues $\Phi _{0}^{e}$ and $\Lambda _{0}^{e}$. The alternative eigenvectors $\Phi ^{e}$ can be written by means of the original eigenvectors and a non-singular matrix *S* [119] as

48 $$\Phi _{0}^{e} =\Phi ^{e} .S$$

Alternatively, to make Eq. (48) more suitable for practical implementation and by using Eq. (46), we can write

49 $$\left[ {\Phi _{{0{\text{R}}}} \Phi _{{ 0 {\text{S}}}} } \right] = \left[ {\Phi _{\text{R}} \Phi _{\text{S}} } \right]\left[ {\begin{array}{*{20}c} {S_{\text{R}} } & {S_{\text{RS}} } \\ 0 & {S_{\text{S}} } \\ \end{array} } \right]$$

By inserting Eqs. (48, 49) into Eq. (47), and by using the orthogonality of eigenvectors, the alternative mass and stiffness matrices of the generic element can be derived as

50 $$M^{e} = M_{ 0}^{e}\Phi _{0} M_{\text{S}}\Phi _{0}^{\text{T}} M_{ 0}^{e} ,\quad K^{e} = K_{0}^{e} \Phi _{{ 0 {\text{S}}}} K_{\text{S}}\Phi _{{ 0 {\text{S}}}}^{\text{T}} K_{0}^{e} ,$$

where

51 $$M_{\text{S}} = S^{\text{T}} S,\quad K_{\text{S}} = S_{\text{S}}^{\text{T}}\Lambda _{\text{S}} S_{\text{S}} ,$$

and Λ_S_ represents the diagonal eigenvalue matrix of the strain modes.

*M*_S_ and *K*_S_ can be varied and used for updating purposes. Moreover, because both matrices involve the modal characteristics, i.e., the eigenvectors and eigenvalues, they can strongly reflect the damaged elements along the FE mesh.

## FE model updating using EAs: damage tracking

In FE model updating for structural damage identification, the residuals of dynamic characteristics are usually combined to represent the deviation of the damaged structure from the initial FE model of the intact structure. On this basis, the objective function commonly consists of one or more suitable residuals of dynamic characteristics as described in Sect. 3. Such an objective function does not benefit any sensitivity-based approach or system matrix-related method [27]. The use of EAs to tackle the optimization problem of FE model updating with the objective function can be, by and large, categorized into two types: single-objective EAs and the multi-objective EAs. The former is used to convert the optimization problem into a single-objective optimization problem using the weighted sum method, and the latter is used to solve the problem directly using the multi-objective optimization paradigm. The basic differences between single-objective EAs and multi-objective EAs can be summarized as follows [52, 129]. In each iteration, a single-objective EA calculates a single-objective function value for each individual in a population of potential solutions, whereas a multi-objective EA evaluates multiple values, simultaneously. Unique optimal solution is obtained by each run of a single-objective EA, while a set of optimal solutions is achieved by a multi-objective EA. When using a combination between a single-objective EA and the weighting sum method to solve multiple objectives, the outcome is a sub-set of the total Pareto optimal solutions, while a powerful multi-objective EA can generate the whole Pareto optimal solutions or at least the majority of them. When solving a multi-objective optimization problem, using single-objective EAs lacks ability to find alternative solutions that trade all conflicting objectives.

### Single-objective EAs

Single-objective EAs can be exploited to solve the optimization problems of FE model updating for damage identification, where the single-objective function is composed of the sum of a set of residuals of dynamic characteristics, linked with weighting factors. The general form of a single-objective function is given as

52 $$\begin{array}{*{20}c} {{\text{minimize}}\,F\left( x \right) = \mathop \sum \limits_{i = 1}^{\text{NF}} w_{i} F_{i} \left( x \right),} \\ {{\text{subject}}\,{\text{to}}\,g_{j} \left( x \right) < 0,\,\, j = 1,2, \ldots ,} \\ {h_{k} \left( x \right) = 0,\,\, k = 1,2, \ldots ,} \\ {X_{\text{L}} \le x \le X_{\text{U}} ,} \\ {\mathop \sum \limits_{i = 1}^{\text{NF}} w_{i} = 1; \,\,w_{i} \ge 0,} \\ \end{array}$$

where *F*_*i*_ is the *i*th residual included in the objective function *F*; *w*_*i*_ denotes the weighting factor for *F*_*i*_; NF is the number of residuals; *h* and *g* signify the constraint functions; *X*_L_ and *X*_U_ are the lower and the upper bounds of *x*, respectively.

Researchers have formulated various single-objective functions by combining two or more residuals of dynamic characteristics. The most frequently used single-objective function is the linear combination of the residual of natural frequencies and that of modal vectors [27, 28, 66, 94]. The linear combination can be defined by merging Eq. (7) and one of equations [8–10]. By way of illustration, several representative single-objective functions are provided as follows.

A typical single-objective function was formulated by Jin et al. [66] in Eq. (53), expressed by

53 $$F = \mathop \sum \limits_{i = 1}^{N} \alpha_{i} \left( {\frac{{\omega_{i}^{\text{I}} - \omega_{i}^{\text{D}} }}{{\omega_{i}^{\text{D}} }}} \right)^{2} + \mathop \sum \limits_{i = 1}^{N} \beta_{i} \frac{{\left( {1 - \sqrt {{\text{MAC}}_{i} } } \right)^{2} }}{{{\text{MAC}}_{i} }},$$

where *F* is the objective function and it includes two parts: the first term is the residual of natural frequencies and the second term is the residual of modal shapes; *N* is number of modes; *α*_*i*_ and *β*_*i*_ denote the weighting factors for the *i*th order natural frequency and the *i*th modal vector, respectively.

Another distinctive objective function was developed by Jung and Kim [27]. They combined the static deflection residual with natural frequency and modal vector residuals as.

54 $$F = \frac{1}{N}\left( {\mathop \sum \limits_{i = 1}^{N} \alpha_{i} \left( {\frac{{\omega_{i}^{\text{I}} - \omega_{i}^{\text{D}} }}{{\omega_{i}^{\text{D}} }}} \right)^{2} + \mathop \sum \limits_{i = 1}^{N} \beta_{i} \sqrt {\frac{{1 - {\text{MAC}}_{ii} }}{{{\text{MAC}}_{ii} }}} } \right) + \frac{1}{M}\left( {\mathop \sum \limits_{j = 1}^{M} \gamma_{j} \left( {\frac{{v_{j}^{\text{I}} - v_{j}^{\text{D}} }}{{v_{j}^{\text{E}} }}} \right)^{2} } \right),$$

where *v*_*j*_ signifies the static deflection measured at the *j*th point; *M* is number of measured deflection points; *γ*_*j*_ is the weighting factors corresponding to the *j*th point’s static deflection, respectively.

Selection of suitable weighting factors to effectively measure the relative importance of each residual is crucial for solving the optimization problem. The weighting factors are usually determined by trial and error.

**Fig. 3:**
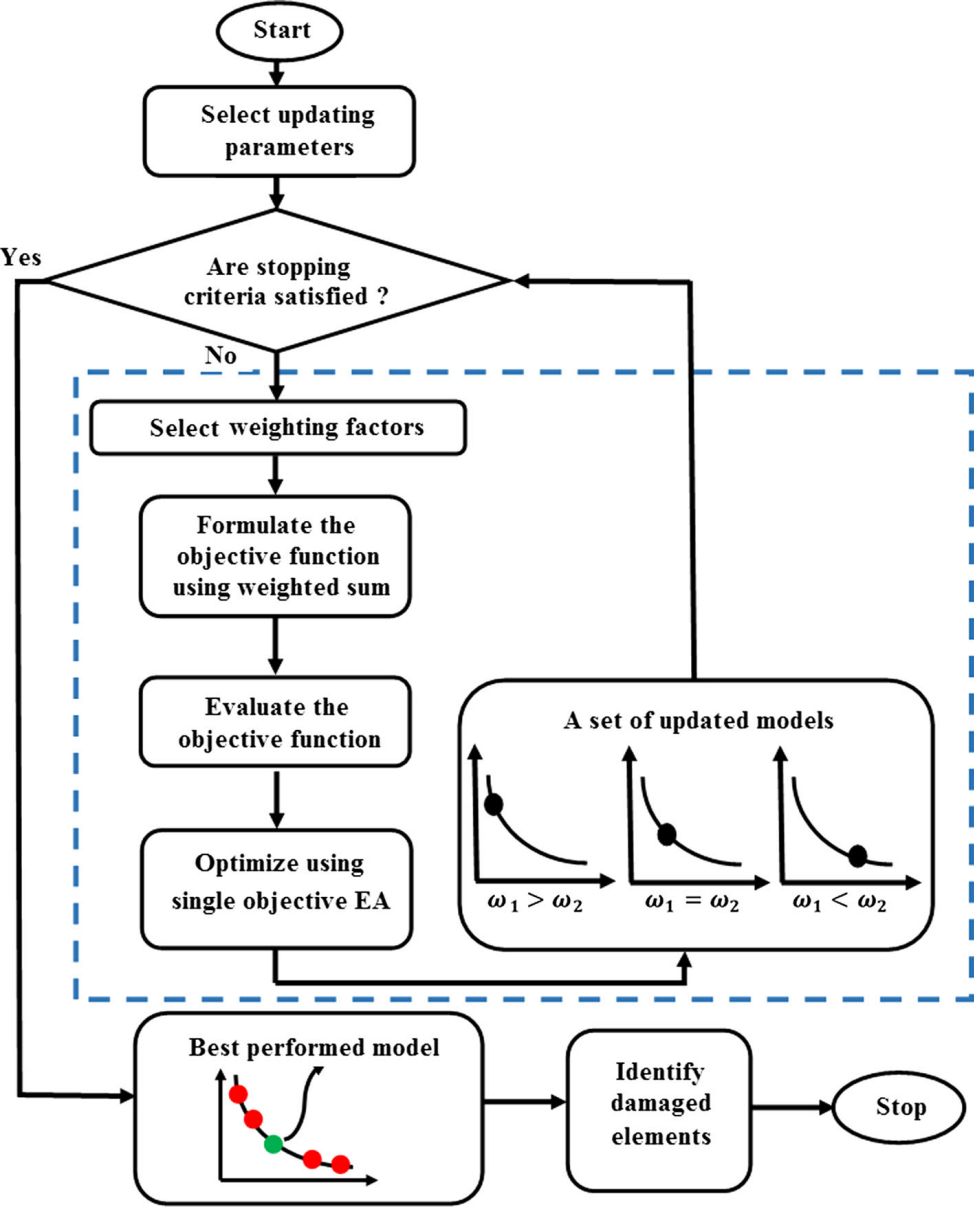
FE model updating using single-objective EAs for damage identification

The procedure of identifying structural damage relying on FE model updating using single-objective EAs is schematized in Fig. 3. In the figure, first, the updating parameters for damage tracking are selected carefully. After checking the stopping criteria, weighting factors are chosen and the objective function is formulated using Eq. (52). Then, a suitable single-objective EA is implemented to create a superior FE model to the initial one. Thereafter, the weighting factors are modified and a new objective function is formulated. When the stopping criteria are satisfied, the process stops and the best performing model is chosen. Finally, the damage patterns are derived by checking the normalizing factors for each element.

FE model updating using single-objective EAs has been used to identify structural damage, where the genetic algorithm (GA) is the most representative method to implement model updating. Xia and Hao [130] utilized a real coded GA to solve FE model updating with the single-objective functions framed by the residual of natural frequencies and that of modal vectors to detect damage in a cantilever beam and a portal frame, with accurate damage identification results obtained. Marwala [28] used the GA to solve FE model updating problem in a damaged asymmetrical H-shaped structure and in a damaged simply supported beam using the residual of modal shapes and that of natural frequency. The GA was found to outperform the Nelder–Mead (NM) simplex method in both applications. Jung and Kim [27] implemented a hybrid GA-NM method for FE model updating on a small-scale bridge, with satisfactory performance of the method proved. Perera and Torres [73] used the GA for tracking damage in a simple beam for various damage cases in noisy conditions. The residual of mode shapes and that of natural frequencies were exploited to form two objective functions for the optimization problem. Their study results showed remarkable ability of the GA in assessing damage based on FE model updating. Au et al. [131] implemented the micro-GA to detect structural damage in both a single-span simply supported beam and a three-span continuous beam using noisy and incomplete modal characteristics. Their observations showed that the damage detection technique was accurate, but noisy modal characteristics negatively affected the results. He and Hwang [132] proposed a hybrid simulated annealing and GA for damage identification in a simple cantilever beam and a clamped beam using displacement-based objective functions.

 Different from the GA, particle swarm optimization (PSO), developed by Kennedy and Eberhart [133], is a typical single-objective EA for FE model updating for structural damage interrogation. Marwala et al. [72] used PSO for FE model updating with the objective function framed by the residual of natural frequencies and that of modal vectors in both damaged asymmetrical H-shaped structure and damaged simply supported beam. The results showed that PSO performed better than GA and simulated annealing (SA) [28]. Moreover, a hybrid NM-PSO [28] outperformed NM and PSO algorithms when they were implemented individually. The superior performance of NM-PSO was attributed to the combined merits of the PSO in global optimization and the NM in local optimization. A compound hybrid algorithm that utilized PSO, ray optimizer, and harmony search, developed by Kaveh et al. [74], was used for damage assessment based on FE model updating in a five-story and four-span frame as well as an A-52-bar space truss, with the robustness for damage detection testified. Saada et al. [78] studied an approach of FE model updating using a modified PSO for damage identification in beams. The proposed method could detect local damage in beams. PSO and modal-based residuals for damage detection in a Timoshenko beam structure were successfully utilized by Gökdağ and Yildiz [134]. An improved PSO was proposed by Kang et al. [135] for damage identification in a simply supported beam and truss structure. Their results showed better performance of the developed method when compared with original PSO, DE, and GA. Seyedpoor [136] proposed a two-stage structural damage detection method using a modal strain energy-based index and PSO. Their method was tested in various structures and showed great performance. Other proved hybrid PSO techniques were investigated in [75–77] for FE model updating procedures.

Other powerful single-objective EAs include differential evolution (DE) [137] cuckoo search (CS) [138], covariance matrix adaptation evolutionary strategy (CMA-ES) [139], and artificial bee colony (ABC) [140]. Seyedpoor et al. [141] proposed a DE and modal characteristics-based framework for damage detection in beams, trusses, and 3D structures. Results showed outstanding performance compared with the results of the PSO. Xu et al. [79] implemented CS on the objective function formed by the residual of modal shapes and that of natural frequencies for damage detection in a dual span simply supported beam and truss structure. Experimental results indicated that the CS was efficient in identifying local damage. The CMA-ES was incorporated into FE model updating to track damage in a quarter-scale two-span reinforced concrete bridge [139], where the single-objective function was created by combining the residual of modal shapes and that of natural frequencies. Ding et al. [142] successfully implemented the ABC with a hybrid search strategy for damage tracking in a 61-bar truss structure and a two-span continuous plate using mode shape and natural frequency residuals. Although the strong ability of the CMA-ES to locate damage was displayed, more experimental validation is needed to further verify this method.

**Fig. 4:**
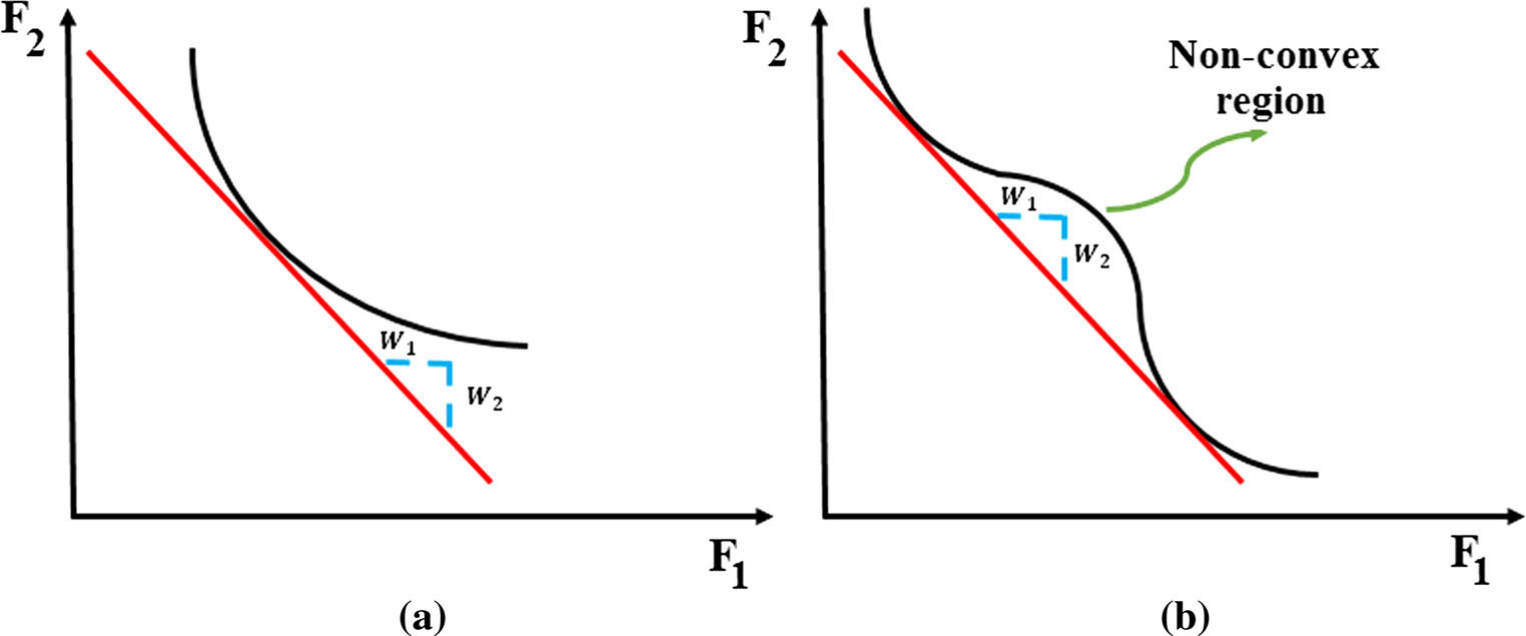
Convex Pareto front (**a**) and non-convex Pareto front (**b**)

Despite the successful applications reported, single-objective optimization using EAs combined with the weighted sum method to solve the detection of damage based on FE model updating has shown some deficiencies. Determination of the optimal weighting factors usually undertaken by trial and error is an exhausting task. It requires the multiple runs of the algorithm while varying the weighting factors at each run. Another problem can arise when the distribution of the Pareto optimal front that contains the set of optimal solutions is non-convex: namely, inability to discover the Pareto solutions in the non-convex region, as shown in Fig. 4. Moreover, the effect of the weighting factors on the objective function is to some extent uncertain: a small variation in one weighting factor can cause a dramatic change in objective function values, whereas a large variation may cause only a small change in those values [52].

### Multi-objective EAs

Multi-objective EAs have stimulated wide interest in solving inverse problems in engineering applications in recent years. Nevertheless, they have rarely been utilized for solving FE model updating problems; in particular, few multi-objective EAs have been concerned with structural damage detection based on FE model updating.

The key to using multi-objective EAs to perform FE model updating for damage detection lies in the formulation of the multi-objective function. In this case, the multi-objective function is formed by substantially combining more than one residual of dynamic characteristic, with no need for linkage by weighting factors [66], expressed as

55 $$\begin{array}{*{20}c} {{\text{minimize }}\left( {F_{i} \left( x \right)} \right) = {\text{minimize}}\left( {F_{1} \left( x \right), F_{2} \left( x \right), \ldots ,F_{NF} \left( x \right)} \right) ,} \\ {{\text{subject}}\,{\text{to}}\,g_{j} \left( x \right) < 0, \quad j = 1,2, \ldots } \\ {h_{k} \left( x \right) = 0, \quad k = 1,2, \ldots } \\ {X_{\text{L}} \le x \le X_{\text{U}} } \\ \end{array}$$

where *F*_*i*_ denotes the *i*th objective function; NF is the total number of objective functions; *g*_*j*_ and *h*_*k*_ signify the constraint functions; *X*_L_ and *X*_U_ imply the lower and the upper bounds of the variable *x*, respectively.

Various multi-objective function formulations have been created with the residuals of dynamic characteristics, similar to those illustrated in Sect. 3. By way of illustration, representative multi-objective functions are explicated as follows.

Kim and Park [94] formulated a multi-objective function:

56 $${\text{min }}\mathop \sum \limits_{i = 1}^{N} \left\{ {\left| {\omega_{i}^{\text{I}} - \omega_{i}^{\text{D}} } \right|, 1 - {\text{MAC}}_{ii} } \right\}$$

where $\omega_{i}^{\text{I}}$ and $\omega_{i}^{\text{D}}$ denote the *i*th natural frequency derived from the initial FE model for an undamaged structure and that from the damaged structure, respectively; MAC_*ii*_ is the diagonal value of the MAC matrix in correspondence to the *i*th mode shape of the initial FE model for the intact structure and that of the damaged structure, respectively.

Jin et al. [66] also developed a multi-objective function using the residual of modal characteristics, as stated in Eqs. (7) and (11), given by

57 $$\hbox{min} \left( {F_{1} = \mathop \sum \limits_{i = 1}^{N} \left( {\frac{{\omega_{i}^{\text{I}} - \omega_{i}^{\text{D}} }}{{\omega_{i}^{\text{D}} }}} \right)^{2} ,\, F_{2} = \mathop \sum \limits_{i = 1}^{N} \frac{{\left( {1 - \sqrt {{\text{MAC}}_{i} } } \right)^{2} }}{{{\text{MAC}}_{i} }}} \right)$$

Perera and Ruiz [95] utilized modal flexibility along with natural frequencies and modal vectors for damage detection based on FE model updating in large-scale structures. They incorporated Eqs. (13) and (29) to design the multi-objective function:

58 $$\hbox{min} \left( {F_{1} = 1 - \mathop \prod \limits_{i = 1}^{N} {\text{MTMAC}}_{i} ,\, F_{2} = 1 - \mathop \prod \limits_{i = 1}^{N} {\text{MACF}}_{i} } \right)$$

Another interesting research study was carried out in [65], where incomplete mode shapes were utilized to derive the residual of MSE, as shown in Eq. (38). The comparison was drawn between the initial (I) and the induced model (IC), without experimental data. Using *S* number of incomplete mode shapes, the objective function was expressed as

59 $$\hbox{min} \left( {\begin{array}{*{20}c} {F_{1} = \mathop \sum \limits_{i = 1}^{{\frac{S}{2}}} \mathop \sum \limits_{j = 1}^{L} \left| {\left( {\varphi_{i}^{\text{I}} } \right)^{\text{T}} K_{j} \left( {\varphi_{i}^{\text{I}} } \right) - \left( {\varphi_{i}^{\text{IC}} } \right)^{\text{T}} K_{j} \left( {\varphi_{i}^{\text{IC}} } \right)} \right|,} \\ {F_{2} = \mathop \sum \limits_{{i = \frac{s}{2} + 1}}^{S} \mathop \sum \limits_{j = 1}^{L} \left| {\left( {\varphi_{i}^{\text{I}} } \right)^{\text{T}} K_{j} \left( {\varphi_{i}^{\text{I}} } \right) - \left( {\varphi_{i}^{\text{IC}} } \right)^{\text{T}} K_{j} \left( {\varphi_{i}^{\text{IC}} } \right)} \right|} \\ \end{array} } \right)$$

where $\varphi_{i}^{\text{I}}$ and $\varphi_{i}^{\text{IC}}$ are the *i*th model vectors of the initial FE model and the induced model, respectively; *K* implies the elemental stiffness matrix; *S* and *L* denote the selected number of incomplete mode shapes and the number of elements in the FE model mesh, respectively.

**Fig. 5:**
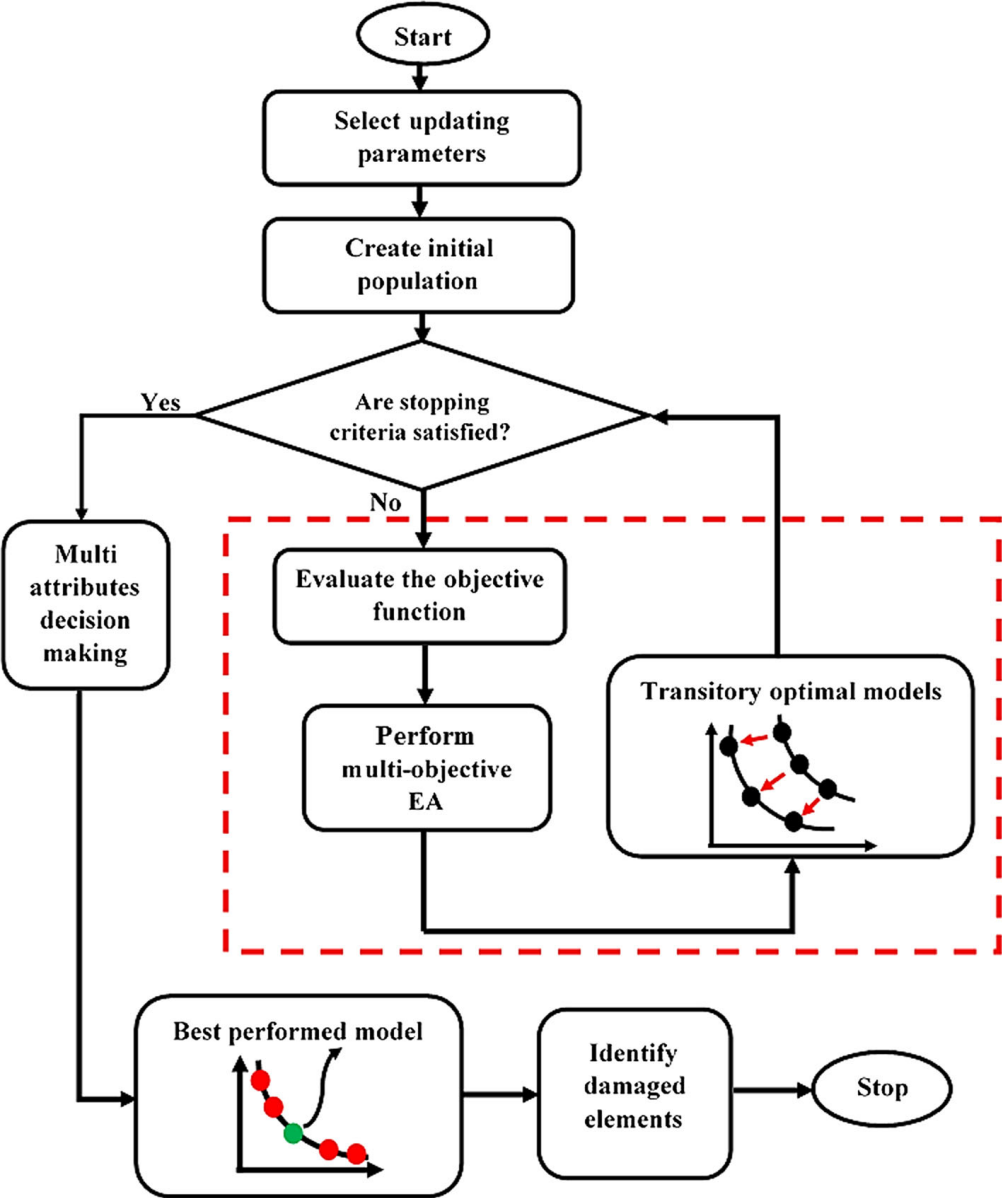
FE model updating using multi-objective EAs for structural damage identification

The procedure of implementing FE model updating using multi-objective EAs to identify structural damage is depicted in Fig. 5. As shown in the figure, the procedure begins with selection of the FE model updating parameters by satisfying the recommendations in Sect. 4, followed by the creation of an initial population of normalizing factors. After that, a complete evaluation of all individuals in the population is performed. Then, the multi-objective EA algorithm is implemented to update the population by eliciting the best FE models for the next generation. When the stopping criteria are satisfied, the procedure progresses to the next step, where a multi-attribute decision-making technique is used to select the best performed model out of the set of the Pareto optimal solutions (models). Finally, the damaged elements are defined by studying the change in normalized factors corresponding to each candidate element in the FE model.

FE model updating using multi-objective EAs has been successfully employed in various damaged structures. Kim and Park [94] solved the FE model updating problem of a plate with a crack using the Pareto GA introduced by Cheng and Li [143], where the appropriate FE model was selected by a criterion relying on the MAC matrix:

60 $$\hbox{min} \,{\text{MAC}}_{ii} > 0.97$$

in which the value of 0.97 indicates high consistency between the two modal vectors of the updated FE model and the damaged structure; in other words, the updated model approximates the damaged structure well.

Kim and Park [94] reported that the proposed multi-objective EA was more efficient than the single-objective EA. Jin et al. [66] tackled the FE model updating problem of a damaged highway bridge using a non-dominated sorting genetic algorithm (NSGA-II), a powerful algorithm introduced by Deb et al. [144]. The NSGA-II incorporates the advantage of GAs with that of non-dominated sorting and crowding distance metric techniques to perform multi-objective optimization. Moreover, they developed a successful multi-attribute decision-making technique to select the optimal FE model from all optimal Pareto front models. They reported that the results obtained from the multi-objective strategy were much superior to those from single-objective optimization using GA. Further, the multi-objective strategy required less computational time and gave more physical meaning to the updated model.

Perera and Ruiz proposed a two-stage updating procedure for damage detection in large-scale structures based on FE model updating. The first stage was identification of potential damage regions by means of damage functions using the method of Teughels et al. [145]. The second stage was the identification of damaged elements in these potential damage regions. During the two stages, the strength Pareto genetic algorithm (SPGA) [146] was applied as a multi-objective EA to recognize the damage region and identify the damage. The results showed that the proposed method was robust, computationally efficient, and could be applied effectively for damage detection in large structures. Cha and Buyukozturk [65] used an implicit redundant representation genetic algorithm (IRR-GA) [147, 148] with NSGA-II [144] to perform multi-objective optimization to solve damage detection of complex 3D structures based on FE model updating. For the selection of the most preferred model, the summation of both parts of the objective function was used. The model with the lowest summation value could be selected as the best model because no trade-off characteristics between the two terms of the objective function had been discovered. Cha and Buyukozturk’s final remarks showed that the proposed method could be used effectively for detecting minor local damage in 3D structures. Although the method successfully detected damage, real-world validation using experimental data is still needed. Wang et al. [149] compared FE model updating using NSGA-II, differential evolution for multi-objectives (DEMO) [150] and multi-objective particle swarm optimization (MOPSO) [151] for damage detection in truss structure. They noted that MOPSO outperformed NSGA-II and MOPSO for all damage patterns.

The summary of implementation of various single-objective EAs and multi-objective EAs and their applications can be listed in Table 1. From the table and above survey, it is observed that most of the existing researches lack comparative studies. Moreover, various EAs have never been tested for the purpose of damage identification in structures using FE model updating with EAs. Also, we notice that not all types of structures have been studied during the applications.

**Table 1:** A summary showing various applications of EAs for structural damage detection with FE model updating

Algorithm	Reported applications	Studies
*Single*-*objective EAs*		
GA and its variations	Various types of beams, bridges, frame structures, and trusses	Jung and Kim [27], Marwala [28], Perera and Torres [73], Xia and Hao [130], Au et al. [131], He and Hwang [132]
PSO and its variations	Various types of beams, bridges, frame structures, and trusses	Marwala et al. [28, 72], Kaveh et al. [74], Saada et al. [78], Gökdağ and Yildiz [134], Kang et al. [135], Seyedpoor [136]
DE	Beams, trusses, and 3D structures	Seyedpoor et al. [141]
ABC	Frames and trusses	Ding et al. [142]
CS	Frames and trusses	Xu et al. [79]
CMA-ES	Bridges	Jafarkhani and Masri [139]
*Multi-objective EAs*		
NSGA-II and its variations	Beams, bridges, and 3D structures	Cha and Buyukozturk [65], Jin et al. [66], Kim and Park [94], Wang et al. [149]
MOPSO	Beams	Wang et al. [149]
DEMO	Beams	Wang et al. [149]
SPGA	Large-scale structures	Perera and Ruiz [95]

## Case study

**Fig. 6:**
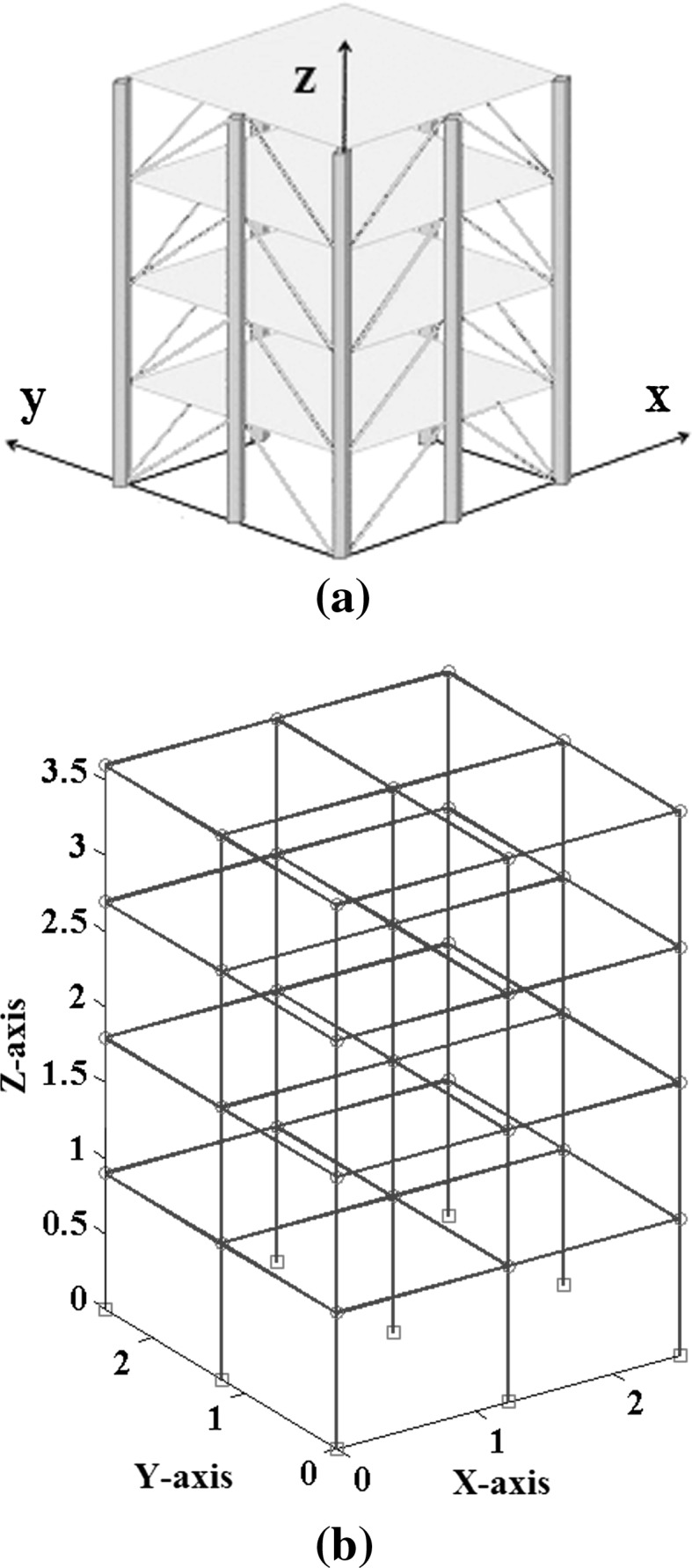
The ASC–ASCE SHM benchmark 4-story building model. **a** The original model [154], **b** the developed model

**Fig. 7:**
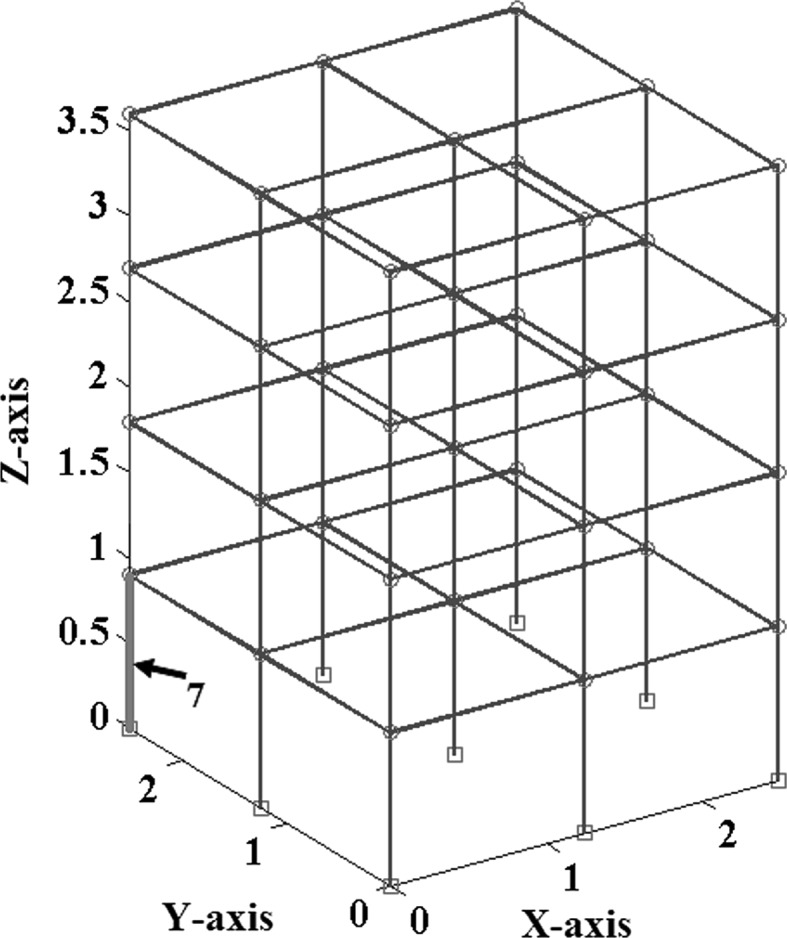
The damage scenario

**Fig. 8:**
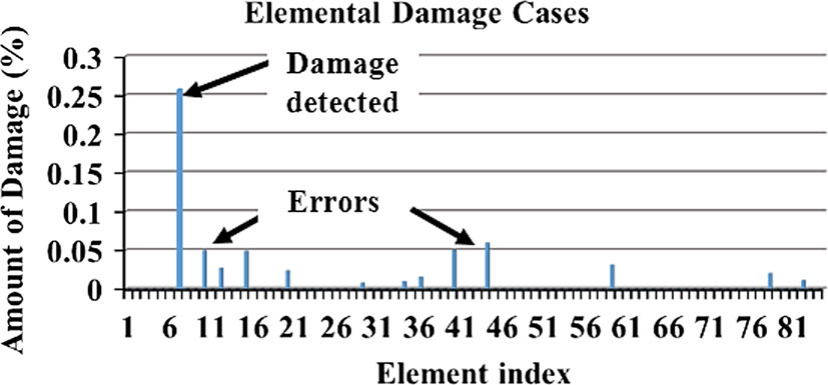
Damage detection using GA

**Fig. 9:**
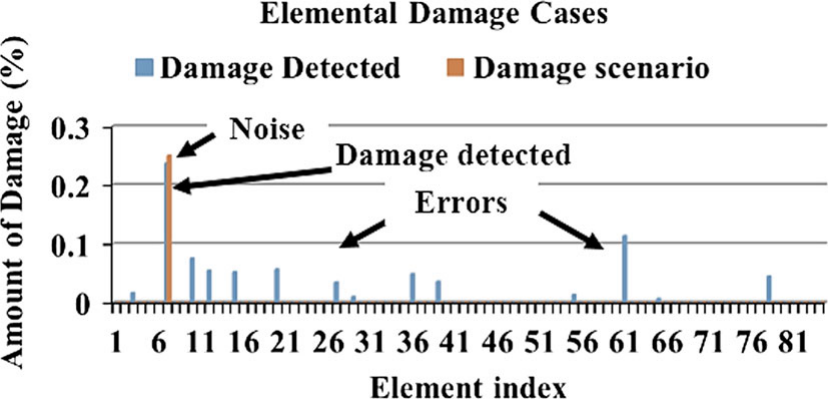
Damage detection using GA under noisy conditions

**Fig. 10:**
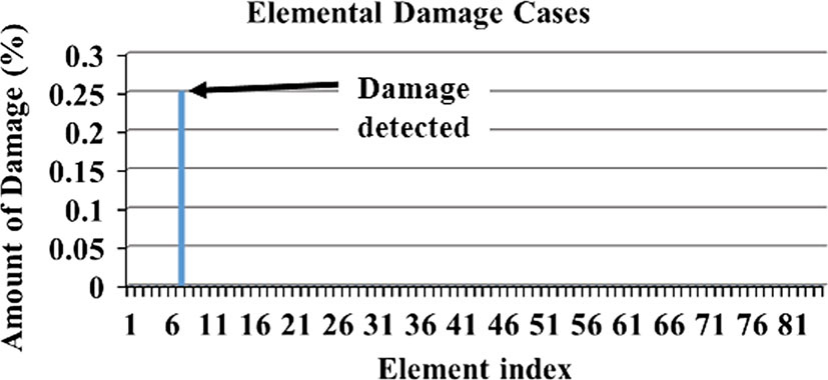
Damage detection using PSO

**Fig. 11:**
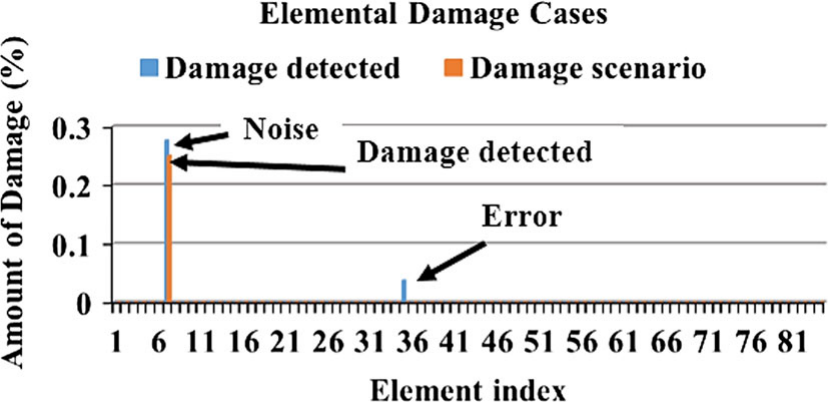
Damage detection using PSO under noisy conditions

**Fig. 12:**
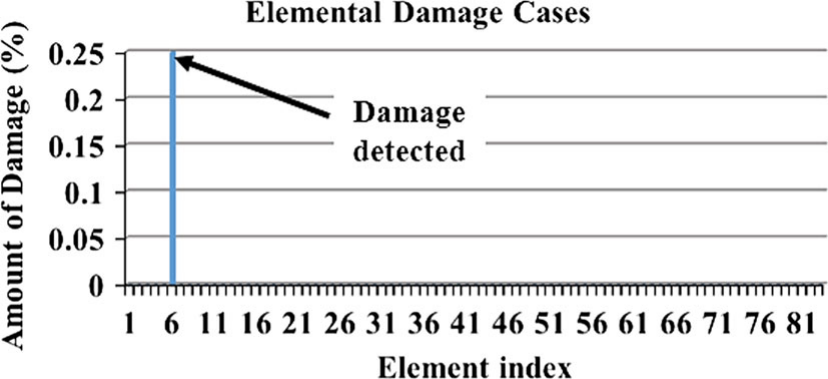
Damage detection using MOPSO

**Fig. 13:**
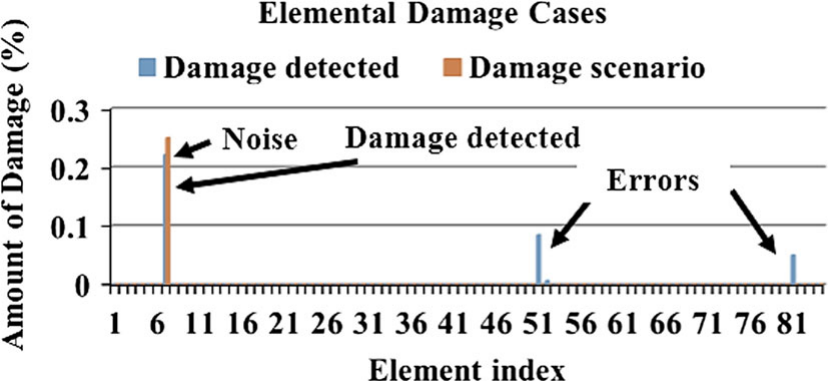
Damage detection using MOPSO under noisy conditions

 In order to make a comparative study between the structural damage identification using FE model updating with single-objective EAs and multi-objective EAs, two single-objective EAs, namely GA [52, 53] and PSO [53, 133], are compared with a multi-objective EA, namely MOPSO [151]. In the application of MOPSO, having a leader solution in each iteration makes a multi-attributes decision-making technique not required. A 3D modular structure is developed based on the Phase II ASC–ASCE SHM benchmark 4-story building [152, 153]. The model is constructed with no side braces along the 4 floors as shown in Fig. 6. This complex model can well illustrate the efficiency of structural damage tracking using FE model updating with EAs. One damage case is considered by simulating damage in element 7 by reducing 25% of its Young’s modulus as in Fig. 7. To simulate noise, ∓5% white noise is added to the mode shapes. The objective function for the optimization problem is formulated by combining the mode shape and the MSE residuals as in Eq. (61). Initially, to execute the three applied algorithms and by following the recommendations reported in [52, 53, 133, 151], various parameter combinations are tested to achieve best performances. Finally, for GA, a population size of 100, tournament selection with size of five individuals, blend crossover with crossover rate of 0.95, and random mutation with mutation probability equal to 0.1 are used. A detailed explanation of GA operators can be observed using a relevant book [52]. Both PSO and MOPSO are implemented by using a population size of 100, inertia factor of 0.5, and acceleration coefficients of 1.2. The velocity vectors are bounded by intervals of [− 0.25, 0.25] to obtain better outcomes. Extra parameters in MOPSO are set as: the number of hypercubes is 10 and the number of individuals in the repository is 50. A complete description of PSO and MOPSO parameters is available in [52, 133, 151]. Results of implementation of GA, PSO, and MOPSO can be seen in Figs. 8, 9, 10, 11, 12, and 13 in “Appendix”. The performances of GA, PSO, and MOPSO are listed in Table 2 by considering the computational time, consistency, and accuracy of results. It is obvious from Table 2 and “Appendix” that GA, PSO, and MOPSO are able to detect damage even under noisy conditions. Nevertheless, PSO has superior performance when compared with GA which in turn results in various estimation errors along the structure. Moreover, MOPSO achieved better performance than GA and similar performance to PSO by means of consistency and reliability.

61 $${\text{Min}}\left( {{\text{MSR}}\left( {\varphi^{\text{I}} ,\varphi^{\text{D}} } \right),{\text{MSER}}\left( {\varphi^{\text{I}} ,\varphi^{\text{D}} ,K} \right)} \right) = {\text{Min}} \left( {\mathop \sum \limits_{i = 1}^{N} \beta_{i} \times (1 - {\text{diag}}\left( {{\text{MAC}}_{i} \left( {\left\{ {\varphi_{i}^{\text{I}} } \right\},\left\{ {\varphi_{i}^{\text{D}} } \right\}} \right)} \right), \mathop \sum \limits_{i = 1}^{N} \left( {\frac{{\varphi_{i}^{{{\text{I}}^{\text{T}} }} K \varphi_{i}^{\text{I}} - \varphi_{i}^{{{\text{D}}^{\text{T}} }} K \varphi_{i}^{\text{D}} }}{{\varphi_{i}^{{{\text{D}}^{\text{T}} }} K \varphi_{i}^{\text{D}} }}} \right)^{2} } \right),$$

where MSR(*φ* ^I^, *φ* ^D^) and MSER(*φ* ^I^, *φ* ^D^, *K*) are the mode shape and the global MSE residuals, respectively; *K* is the global stiffness matrix; MAC_i_ is the MAC value corresponding to the *i*th mode shape; *φ* ^I^ and *φ* ^D^ are the mode shapes obtained from the initial FE model of the undamaged structure and those from the damaged structure; diag(MAC) is the *i*th diagonal element of the MAC matrix; *β*_*i*_ is a weighting factor defining the relative importance of MAC_*i*_; *N* is the total number of mode shapes.

**Table 2:** A performance comparative study between the applied EAs

Algorithm	Mean computational cost (s)	Consistency	Accuracy (minimum objective function value)	
			Damage without noise	Damage with noise
GA	795	6	11.2 × 10^−4^	0.08
PSO	345	9	3.07 × 10^−8^	0.072
MOPSO	564	8	3.57 × 10^−7^	0.0722

In order to suggest possible future research directions and from the case study, it is important to conduct more comparative studies on the application of various existing EAs to recommend the most efficient algorithms. Moreover, it is necessary to study different types of structures during the structural damage detection procedure. Finally, the existence of many dynamic characteristics can influence more research about the application of various residuals of dynamic characteristics in order to formulate powerful objective functions able to transfer damage information efficiently.

## Concluding remarks

This paper surveyed the technologies of FE model updating using EAs and their applications in damage detection. A theoretical background addressed the structural damage detection problem, and FE model updating methods were illustrated. The common dynamic characteristics employed to develop residuals used in formulating the objective functions for damage tracking were investigated. The uses of single-objective EAs and multi-objective EAs for damage identification via FE model updating were evaluated. Finally, a case study showed the applications of FE model updating-based structural damage detection with two single-objective and one multi-objective EAs were conducted. This survey suggests several potential research directions to further enhance the use of FE model updating using EAs for structural damage detection:

- Most relevant studies have focused on applications of FE model updating using EAs for damage identification in small- and medium-scale structures such as beams, frames, 2D structures. For that reason, it is recommended to make more efforts to apply EAs to solve damage detection in large-scale complex structures using FE model updating.
- Although various combinations of dynamic characteristics have been used in the formulation of the objective function, there is a trend to solve complex damage assessment based on the FE model updating problem using EAs by combining other different dynamics characteristics to form the objective function.
- Many powerful EAs available in the literature have never been implemented to solve the damage detection problem based on FE model updating. Therefore, it is worth exploring the most suitable EAs to achieve accurate and reliable results of damage identification as well as considering computational efficiency.
- The various types of EAs with different features and application scopes should stimulate more comparative studies in order to define the most applicable and reliable algorithms for structural damage identification using FE model updating.
- Noisy measurements and incomplete data are major issues facing damage detection based on FE model updating. Most of the existing studies verified their techniques by either computer simulations or laboratory experiments. Hence, industrial implementations have to be carried out to study the effects of those issues.
- Multi-attribute decision-making techniques are essential for determining trade-off solutions from the overall solutions in the Pareto front when using multi-objective EAs. Such techniques identify the most adequate solution to be chosen for damage tracking. To date, only a few related applications exist in the field of FE model updating.

## Appendix

See Figs. 8, 9, 10, 11, 12, and 13.
